# Active autophagy in the tumor microenvironment: A novel mechanism for cancer metastasis

**DOI:** 10.3892/ol.2012.1015

**Published:** 2012-11-07

**Authors:** YINGHUA XU, XIAOPING XIA, HONGMING PAN

**Affiliations:** 1 Departments of Oncology, Sir Run Run Shaw Hospital, School of Medicine, Zhejiang University, Hangzhou, Zhejiang 310016, P.R. China; 2 Clinical Laboratory, Sir Run Run Shaw Hospital, School of Medicine, Zhejiang University, Hangzhou, Zhejiang 310016, P.R. China

**Keywords:** autophagy, microenvironment, metastasis, pathway, cancer

## Abstract

Autophagy is a lysosomal degradation process which is key for the regulation of the turnover of long-lived or damaged proteins and organelles and which promotes cell survival during nutrient deprivation or other microenvironmental stresses. Current evidence supports the hypothesis that autophagy suppresses tumorigenesis, particularly during the early stages of tumor initiation. However, in established tumors, autophagy promotes survival under stressful conditions during cancer progression and in response to chemotherapy; however, the mechanism by which autophagy influences cancer metastasis remains unknown. In this review, we discuss the capacity of an abnormal tumor environment to induce autophagy and consider how this relates to tumor metastasis and the attractive prospect of manipulating autophagic signaling pathways as potential targets for the treatment of cancer metastasis.

## Contents

1. Introduction
2. Active autophagy in tumor microenvironment and cancer cell fate
3. Hypoxia and anoxia
4. Nutrient deprivation
5. ECM detachment
6. ER stress
7. Autophagy induced by tumor microenvironmental stresses and tumor metastasis
8. Manipulating autophagy induced by tumor microenvironmental stresses for cancer therapy
9. Conclusions/perspectives

## 1. Introduction

Autophagy is an evolutionarily conserved catabolic process in which intracellular membrane structures sequester proteins and organelles to degrade and turn over these cytoplasmic constituents; thus, it is essential for growth regulation and the maintenance of homeostasis (1–3). Autophagy is a multi-step process characterized by nucleation, elongation and autophagosome and autolysosome formation, and is tightly regulated by a limited number of highly conserved genes called autophagy regulators (ATGs) (4,5). Defective autophagy is correlated with diverse pathologies, including neurodegeneration, liver, heart and muscle diseases, ageing, inflammation and cancer (6).

Autophagy is activated in response to multiple stresses during cancer progression, including hypoxia, nutrient deprivation, extracellular matrix (ECM) detachment, endoplasmic reticulum (ER) stress and other diverse stresses (7,8). Autonomous proliferating cancer cells are often exposed to conditions such as hypoxia or/and nutrient deprivation, so there must be an alternative metabolic pathway to protect tumor cells from these environmental stresses (9). Moreover, in order to metastasize, tumor cells must adapt to a stressful microenvironment as they disseminate into the systemic circulation and colonize distant organ sites (10). Therefore, when environmental stresses emerge, tumor cells are able to catabolize existing cytoplasmic components to provide essential ingredients to maintain survival by autophagy (11).

Autophagy facilitates cellular survival by enabling cancer cells to grow under stressful conditions. The enhancement of autophagy leads to degradation of proteins and organelles to provide amino acids, fatty acids and nucleotides for reuse (12). It is increasingly appreciated that autophagy provides cancer cells with certain selective advantages in response to various stresses in the primary tumor microenvironment as well as the microenvironment during dissemination and metastasis (13). Paradoxically, however, in certain cases autophagy also contributes to the death of cancer cells by scavenging damaged oxidative organelles (14). In this review, we argue that understanding the net effect of autophagy on enabling cells to cope with diverse stresses of the microenvironment, and thereby controlling the fate of cancer cells and metastasis, may develop new therapeutic strategies based on the regulation of autophagy.

## 2. Active autophagy in tumor microenvironment and cancer cell fate

**Figure 1.**
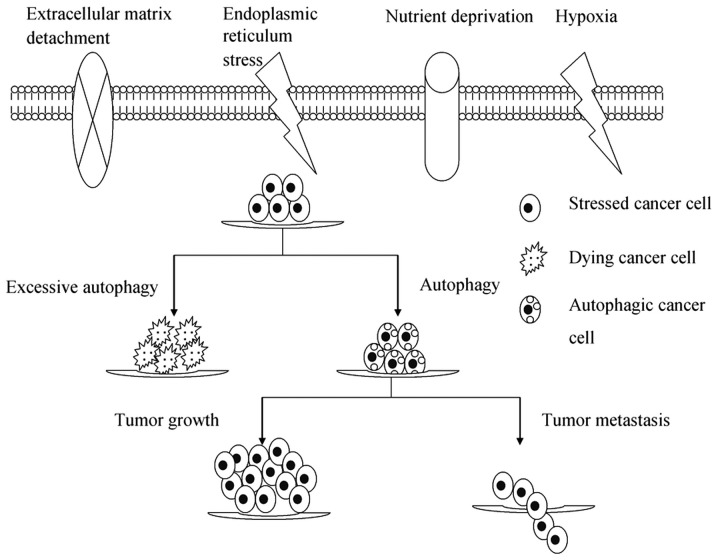
Tumor microenvironmental stresses induce autophagy and affect cancer cell growth and metastasis. Autophagy is activated in response to multiple stresses during cancer progression, including hypoxia, nutrient deprivation, extracellular matrix detachment, endoplasmic reticulum stress and other stresses. Under these stressful conditions, autophagy constitutes a major protective mechanism that allows cells to survive in the primary tumor and migrate into adjacent connective tissue, leading to metastasis in carcinomas. However, if microenvironmental stresses persist, excessive autophagy may ultimately lead to autophagic cell death.

Microenvironmental stresses, as a result of either insufficient oxygen/nutrient supply or increased energetic demands of rapidly dividing tumor cells, induce autophagy as an alternative source of energy and metabolites to ensure that cell growth is appropriate to the environmental conditions (15). Increasing evidence suggests that autophagy constitutes a major protective mechanism that allows cells to survive in response to multiple stresses, including hypoxia, nutrient deprivation, ECM detachment and ER and other stresses (15–17). However, if microenvironmental stresses persist, excessive autophagy may ultimately lead to autophagic cell death, termed type II-programmed cell death (Fig. 1).

## 3. Hypoxia and anoxia

**Figure 2.**
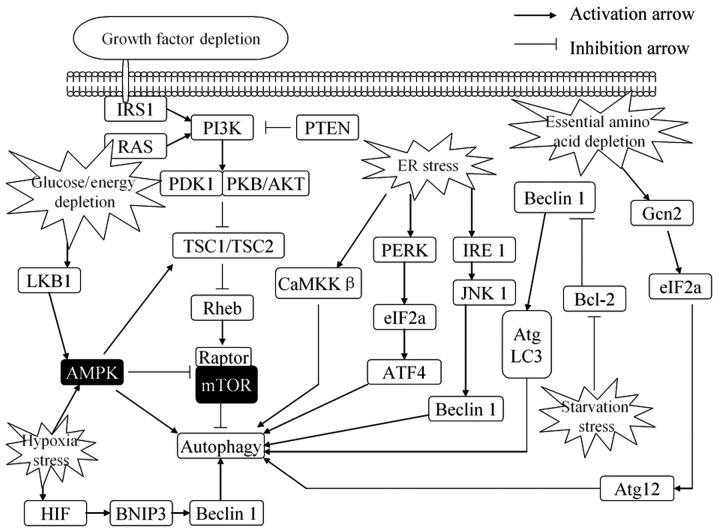
Regulation of autophagy in response to stress. Autophagy is activated in response to multiple stresses during cancer progression, including nutrient deprivation, ER stress, hypoxia, glucose/energy depletion and other diverse stresses. ER stress stimulates autophagy through the PERK-eIF2α pathway, IRE1-JNK1 pathway and Ca^2+^ release. Growth factors, through AKT-dependent and ERK-dependent phosphorylation, suppress autophagy. Depletion of nutrients or energy (amino acids, glucose, energy or serum) induces autophagy by activating the AMPK pathway or promoting upregulate transcription of certain autophagy genes. Autophagy is also induced by hypoxia that signals via AMPK to inhibit mTOR activity or disrupt the Bcl-2-Beclin 1 interaction and activate Beclin 1. Conversely, autophagy is inhibited by increased growth factor signaling through the activation of the Class I group of PI3-kinases and Akt to promote mTOR activity. ER, endoplasmic reticulum; ERK, extracellular signal-regulated kinase; AMPK, AMP-responsive protein kinase; ATF4, activating transcription factor 4; mTOR, mammalian target of rapamycin; BNIP3, Bcl-2/adenovirus E1B 19 kDa-interacting protein; ATG, autophagy regulator.

Hypoxia and anoxia (with oxygen concentrations <3% and <0.1%, respectively) induce autophagy through a variety of different mechanisms (18). Enhanced autophagy is frequently observed in hypoxic regions of solid tumors caused by inadequate vascularization and contributes to cell survival (19). These hypoxic regions are considered to be associated with altered cellular metabolism and poor prognosis. The main transcription factors mediating the hypoxic response are hypoxia-inducible factors (HIFs), which modulate tumor cell metabolism, angiogenesis, growth and metastasis (20). Bcl-2/adenovirus E1B 19 kDa-interacting protein (BNIP3), a BH3-only protein, is a downstream target of HIF-1α and has been shown to induce autophagy by disrupting the Beclin 1-Bcl-2 complex and releasing Beclin 1 in response to a hypoxic microenvironment (21,22). BNIP3L (BNIP3-like protein, also known as NIX), another HIF-1-induced target, is also important for targeting the mitochondria to autophagosomes for clearance (23). Further study has revealed hypoxia- and oxidative stress-mediated activation of the HIF-1α and NFκB pathway in fibroblasts, thereby driving the autophagic flux to promote tumor cell survival (24). HIF-2 is also a potent regulator of chondrocyte autophagy and this protein acts as a brake to the stimulatory function of HIF-1 (25). Recently, the epidermal growth factor receptor antibody cetuximab was found to induce autophagy in cancer cells by downregulating HIF-1α and Bcl-2 and activating the Beclin 1/hVps34 complex (26). In addition, several distinct oxygen sensing pathways that regulate the cellular response to hypoxia have been defined, including activation of the unfolded protein response (UPR), inhibition of the mammalian target of rapamycin (mTOR) kinase signaling pathway and activation of AMP-responsive protein kinase (AMPK), which are all associated with the induction of autophagy (Fig. 2) (27,28). Although hypoxia-driven tumor metabolism and autophagy have been demonstrated, a more detailed mechanism of the interaction between autophagy and a hypoxic tumor microenvironment remains to be determined.

## 4. Nutrient deprivation

Proliferating cancer cells require continuous access to resources that sustain intracellular energy and nutrient levels, but the tumor microenvironment is not sufficient to supply these essential ingredients for cancer cell survival (29). Under these conditions, cancer cells are likely to encounter a shortage of nutrients; therefore, cancer cells must seek alternative metabolic processes to cope with this stress and maintain their survival. Studies have shown that autophagy plays a critical role in protecting cells against a shortage of nutrients by removing damaged substrates for recycling, but the exact mechanism by which cancer cells obtain energy sources under conditions in which their external nutrient supply is extremely limited remains unclear (30,31).

Nutrient (including amino acids and glucose) depletion is the most potent known physiological inducer of autophagy. Ammonia, generated from glutamine deamination in mitochondria, was found to function as an autocrineand/or paracrine-acting stimulator of autophagic flux (32). Autophagosomes were actively induced and promptly consumed in colorectal cancer cells under amino acid- and glucose-deprived conditions, which may contribute to the survival of the cancer cells in their microenvironment (29). Glucose deprivation may cause oxidative stress and stimulate autophagy (33). mTOR and AMPK have been best characterized as critical signaling pathways regulating nutrient deprivation-induced autophagy (Fig. 2) (25,34). Autophagy is also triggered to protect cancer cells from nutrient deprivation by activation of AMPK (35). A previous study has suggested that ubiquilins also accelerate autophagosome maturation and promote cell survival during nutrient starvation (36). The cellular amino acids, especially branched chain amino acids, are a crucial upstream component for the functional activation of mTORC1. The absence of amino acids induces autophagy through the regulation of mTOR activity (Fig. 2) (37). In addition to amino acids, cells must also be supplied with glucose to maintain a constant supply of ATP; during a lack of glucose, autophagy is often activated to maintain intracellular energy homeostasis (38,39). Moreover, it has been reported that the receptor for advanced glycation end products (RAGE) sustains autophagy and limits apoptosis by inhibiting mTOR, resulting in the promotion of pancreatic tumor cell survival (40). Overall, autophagy constitutes a major protective mechanism that allows cells to survive nutrient deprivation.

## 5. ECM detachment

Integrin-mediated attachment of epithelial cells to the ECM is vital for cell growth and survival (41). The loss of ECM attachment leads to apoptosis, termed anoikis (42). However, previous studies have shown that a lack of appropriate matrix contact also robustly induces autophagy to promote cell survival, either during early carcinoma formation or in the later stages of dissemination and metastasis (43,44). Moreover, ECM components modulate autophagy and mitigate its role in cell survival. In HeLa cells, the mechanism by which this occurs has been shown to be dependent on the adhesion of the cells to collagen I or IV (45). In a three-dimensional (3D) culture system using MCF10A mammary epithelial cells grown in low ECM attachment conditions, autophagy was rapidly induced to enhance cell survival during anoikis (46). Although the intracellular signals linking ECM detachment to autophagy remain unclear, the results suggest that autophagy may be a previously unrecognized mechanism which enhances the survival of tumor cells lacking proper ECM contact.

## 6. ER stress

The ER is an organelle responsible for crucial biosynthetic and signaling functions in eukaryotic cells (47). Dysfunction of ER or ER stress may result from various disturbances, including hypoxia and oxidative stress, which elicit a cellular stress response known as the UPR (48). The UPR initially serves as an adaptive mechanism to maintain ER homeostasis. However, severe or prolonged ER stress also switches the cytoprotective functions of UPR and autophagy into cell death, usually by activating intrinsic apoptosis (49).

It has been recognized that in order to clear the accumulation of terminally misfolded protein aggregates that cannot be degraded by the proteasome, the UPR may upregulate the autophagy machinery (50). Activating transcription factor 4 (ATF4) has been shown to facilitate autophagy through direct binding to a cyclic AMP response element binding site in response to ER stress (51). Activation of AMPK by atorvastatin enhances p21 expression and ER stress response, leading to autophagy, which promotes the survival of cancer cells (52). Autophagy may also eliminate a specific type of misfolded procollagen and play a protective role in cell survival against ER stress (53). By contrast, persistent ER stress also induces cell death by activating apoptosis. Cannabinoid action induces autophagy-mediated cell death through stimulation of ER stress in human glioma cells (54). Moreover, the ER stress activates radiation-induced autophagy by PERK-eIF2α in caspase-3/7-deficient cells, which promotes radiosensitivity *in vitro* and *in vivo*(55). It has been demonstrated that ER stress-induced cell death was mediated by autophagy (56), which was partly attributed to the inactivation of AKT/TSC/mTOR (Fig. 2). As discussed above, it is clear that ER stress and autophagy are capable of activating prosurvival mechanisms as well as lethal programs, but the specific mechanisms linking UPR to autophagy during ER stress remain poorly understood.

## 7. Autophagy induced by tumor microenvironmental stresses and tumor metastasis

Tumor microenvironmental stresses have recently gained much attention as a critical determinant of tumor progression since autophagy is often induced as a major protective mechanism that allows cells to survive in response to these stresses. In addition, some clinical evidence suggests that autophagy is used as a survival strategy by established tumors to promote tumor progression.

Autophagy may promote metastasis by enhancing tumor cell fitness in response to microenvironmental stresses. Pancreatic cancer remains a devastating and poorly understood malignant cancer and hypoxia in pancreatic cancers is known to increase malignant potential. In the peripheral area of pancreatic cancer tissue, high expression of LC3, a key component of autophagy, is correlated with poor overall survival and a shorter disease-free period (57). Recent study has also suggested that high expression of the autophagy-related Beclin 1 protein predicts poorer overall survival, progression-free survival and distant metastasis-free survival for nasopharyngeal carcinoma patients (58). The microtubule-associated protein 1 light chain 3 (LC3A) is an essential component of the autophagic vacuoles and LC3A immunohistochemistry renders three patterns of autophagic expression in breast carcinomas: diffuse cytoplasmic, perinuclear and ‘stone-like’ intracellular structures (SLS). Perinuclear LC3A accumulation in colorectal tumour cells is a marker of good prognosis, while high SLS counts were associated with metastases and poor prognosis (59). Phospho-enriched protein in astrocytes (PEA-15) is a 15-kDa phosphoprotein that induces autophagy in human ovarian cancer cells and is associated with prolonged overall survival (60). γ-aminobutyric acid type A (GABAA) receptor-associated protein (GABARAP), the mammalian homolog of yeast Atg8, is involved in autophagosome formation during autophagy and is a new independent prognostic marker for colorectal carcinoma and the overexpression of this protein is associated with poor differentiation as well as shortened overall survival in colorectal cancers (61).

Conversely, autophagy may also inhibit metastasis. Beclin 1 and LC3, crucial genes for autophagy, are altered in several types of human cancer. A higher level of Beclin 1 expression is strongly associated with longer survival of colon cancer patients with stage IIIB disease (62). Autophagy-active Beclin 1 has also been shown to be significantly correlated with the survival of non-Hodgkin lymphoma patients (63). Moreover, Beclin 1 and LC3 significantly decrease with melanoma progression (64). Beclin 1 may play a role in the inhibition of the development of breast cancer and this inhibition may be due to an interaction with Bcl-2 protein and inactivation of PI3K/PKB signaling pathway (65,66). The high expression level of Beclin 1 protein has been demonstrated to be positively correlated with apoptosis and negatively with cell proliferation in gliomas (67). Beclin 1 defects caused by the overexpression of Bcl-xL may facilitate tumor malignant differentiation, which results in a more aggressive cancer cell phenotype and poor prognosis of hepatocellular carcinoma (68). Low Beclin 1 expression is associated with worse overall survival and progression-free survival in extranodal natural killer T-cell lymphoma (69).

Although these proteins have been used to detect and measure levels of autophagy in human tumor samples, few may be universally and accurately applied for autophagy detection in clinical samples. Consequently, there is a rapidly growing need for exploiting ‘gold standard’ for methods and better markers to monitor autophagic activity (70).

## 8. Manipulating autophagy induced by tumor microenvironmental stresses for cancer therapy

As discussed above, cancer cells gain survival and proliferation advantages by autophagy to cope with micro-environmental stresses. Despite the determination of the survival-promoting role of autophagy, it is also well recognized that elevated and/or prolonged autophagy may result in cell death. Therefore, inhibiting autophagy induced by tumor microenvironmental stresses or enhancing excessive microenvironmental stresses to give rise to autophagic cell death may be a promising strategy for cancer therapy. Based on the correlation between microenvironmental stresses and autophagy, certain chemotherapeutic agents and antineoplastic therapies have been reported as an adjuvant therapy for cancer, including acid sphingomyelinase (71), thiazolidinediones (72), tetraspanin (73), bortezomib (74), Δ(9)-tetrahydrocannabinol (54), etformin (75), 2-deoxyglucose (76) and the arginine deiminase ADI-PEG20 (77). However, this therapy has not been further explored for clinical application. In order to accelerate this clinical application, large-scale and multicenter collaboration are necessary.

## 9. Conclusions/perspectives

Autophagy is a catabolic adaptive process usually activated in response to adverse microenvironmental stresses which may have either a beneficial or detrimental cellular effect, depending on the response to environmental stresses (78,79). Currently, it is becoming clear that autophagy is a survival pathway that enables tumor cells to survive under stressful conditions, including hypoxia, nutrient deprivation, ECM detachment and ER stress. By contrast, prolonged activation of autophagy may lead to cell death by cellular self-degradation (80–82).

The tumor environment is a complex and highly dynamic environment, playing a central role in controlling tumor cell behavior and metastasis formation (83). Reduced levels of oxygen and nutrients and malfunction of ECM and ER are critical parameters modulating the tumor microenvironment. As discussed above, abnormality in the tumor microenvironment induces autophagy to aid the maintenance of cancer cell viability and promote cancer cell metastasis under these stressful conditions. However, in certain cases autophagy also contributes to cancer cell death and inhibits metastasis. Based on the functional correlation between microenvironmental stresses and autophagy, a number of new cancer therapeutics have been exploited, but certain limitations prevent widespread clinical application. First, the question of whether we should try to enhance or inhibit autophagy in cancer treatment is not straightforward since it is unclear how autophagic cell death is distinguished from autophagy during cell survival. The engulfment receptor Draper was found to be the first factor that distinguishes autophagy associated with cell death from that associated with cell survival (84). This finding is especially critical since numerous current cancer therapeutics activate or inhibit autophagy, although Draper has not been applied to cancer research. Second, to maximize the potential to be applied for more stringent clinical study, characteristics of methods and better markers to monitor autophagic activity may need to be examined. Third, published studies concerning antineoplastic therapies based on the correlation between the autophagy and tumor microenvironment are short of high-level clinical evidence. Large-scale and multicenter collaborations are necessary in the future. Finally, the molecular mechanisms that underlie autophagy induced by multiple tumor microenvironmental stresses and cancer metastasis remain to be determined.

## Abbreviations:

ATGs: autophagy regulators
AMPK: AMP-responsive protein kinase
ATF4: activating transcription factor 4
BNIP3: Bcl-2/adenovirus E1B 19 kDa-interacting protein
BNIP3L: BNIP3-like protein
ECM: extracellular matrix
HIFs: hypoxia-inducible factors
mTOR: mammalian target of rapamycin
SLS: ‘stone-like’ intracellular structures
UPR: unfolded protein response
